# Through the wall human heart beat detection using single channel CW radar

**DOI:** 10.3389/fphys.2024.1344221

**Published:** 2024-01-24

**Authors:** Sourav Kumar Pramanik, Shekh Md Mahmudul Islam

**Affiliations:** Department of Electrical and Electronic Engineering, University of Dhaka, Dhaka, Bangladesh

**Keywords:** single channel CW radar, maximal overlap discrete wavelet transform (MODWT), heart rate, through-the-wall, fast fourier transform (FFT)

## Abstract

Single-channel continuous wave (CW) radar is widely used and has gained popularity due to its simple architecture despite its inability to measure the range and angular location of the target. Its popularity arises in the industry due to the simplicity of the required components, the low demands on the sampling rate, and their low costs. Through-the-wall life signs detection using microwave Doppler Radar is an active area of research and investigation. Most of the work in the literature focused on utilizing multi-channel frequency modulated continuous wave (FMCW), CW, and ultra-wideband (UWB) radar for their capability of range and direction of arrival (DOA) estimation. In this paper, through-the-wall single-subject and two-subject concurrent heart rate detection using single-channel 24-GHz CW radar leveraged with maximal overlap discrete wavelet transform (MODWT) is proposed. Experimental results demonstrated that the repetitive measurement of seven different subjects at a distance of 20 cm up to 100 cm through two different barriers (wood and brick wall) showed an average accuracy of heart rate extraction of 95.27% for varied distances (20–100 cm) in comparison with the Biopac ECG acquisition signal. Additionally, the MODWT method can also isolate the independent heartbeat waveforms from the two subjects’ concurrent measurements through the wall. This involved four trials with eight different subjects, achieving an accuracy of 97.04% for a fixed distance of 40 cm from the Radar without estimating the angular location of the subjects. Notably, it also superseded the performance of the direct FFT method for the single subject after 40 cm distance measurements. The proposed simpler architecture of single-channel CW radar leveraged with MODWT has several potential applications, including post-disaster search and rescue scenarios for finding the trapped, injured people under the debris, emergency evacuation, security, surveillance, and patient vital signs monitoring.

## 1 Introduction

Through-the-wall vital signs monitoring of human subjects using Doppler radar is an active area of research and gaining attention to the scientific community for its non-contact and unobtrusive form of measurement (Harikesh et al., 2021). The Doppler radar transceiver sends a continuous wave (CW) signal toward the subject, and the reflected signal is phase-modulated by the subject’s periodic tiny movement of the chest surface (Li et al., 2021). The phase of the reflected signal carries information about the subject’s vital signs including heart rate (Cho and Park, 2018; Li et al., 2021). Many studies have demonstrated that Doppler radar may be employed in post-disaster search and rescue applications where the detection of heartbeat through the wall is necessary (Chen et al., 2000).

Prior reported research attempts mostly focused on exploring ultra-wideband (UWB) and frequency-modulated continuous wave (FMCW) radar for the detection of the respiration rate through the wall (Lubecke et al., 2007; Wang et al., 2012; Alizadeh et al., 2019; Duan and Liang, 2019). In (Liu et al., 2016; Xu et al., 2019), a wideband chaotic signal was proposed to detect the range and respiratory signal of a human subject. In Liu et al. (2016), a correlation-based technique is used to detect life signs from echo signals. The composite signal, which combines a wideband chaotic signal with an embedded single-tone signal, is delivered concurrently, and the system can identify respiration, heartbeat, and target localization from the received signal (Liu et al., 2016). In Liu and Liu (2014), stepped frequency radar is employed to monitor vital signs, whereas in Wang et al. (2014a), a linear FMCW idea is applied. In Wang et al. (2014b), a hybrid radar with FMCW and interferometry modes is proposed for vital sign monitoring. Range information is collected using FMCW mode, while chest movement is detected using interferometry mode. The suggested radar can continuously track and monitor human subjects. The FMCW radar at 24 GHz was discussed in Will et al. (2019) to monitor and detect human subjects but not their vital signs. However, because of the complicated architectures of UWB and FMCW, these systems are expensive, signal processing is challenging, and the larger bandwidth utilized with these systems results in diminished system sensitivity (Harikesh et al., 2021).

CW radar has a simpler architecture than FMCW and UWB radar but it can not estimate the range of the target because of the continuous transmission of the electromagnetic signal (Harikesh et al., 2021). Biomedical Doppler radar system has two receiver output channels, one is in-phase (*I*) and another one is quadrature-phase (*Q*). The advantage of using a quadrature receiver is that it fixes the null point problem the single-channel radars have (Park et al., 2006). Null points refer to the parts where the baseband signal is no longer directly proportional to the tiny chest movements causing large errors in the detection of breathing and heart signals (Park et al., 2006). Null points problem in a single-channel radar transceiver can be avoided by adjusting the distance from the radar module to the subject (Park et al., 2006). In Doppler radar transceivers, quadrature receivers are employed to prevent the phase demodulation null points problem (Yavari et al., 2015). Channel imbalance generates large inaccuracies in displacement measurement (Yavari et al., 2015) and changes the forms of physiological signals, leading to errors in the effective radar cross-section and breathing rate estimate in Doppler radar physiological monitoring (Yavari et al., 2015; Kim and Kim, 2019). Single-channel receiver architectures can solve this problem, however, they necessitate complicated coherent signal production and higher sampling rates (Yavari et al., 2015; Kim and Kim, 2019).

Recently, one study demonstrated that two different CW frequencies (2.45 and 2.49 GHz) were transmitted and received simultaneously to detect the distance between the radar and the human target through the wall (Harikesh et al., 2021). In practical applications, estimating the range of the target using two different horn antennas for transmitting two different frequency signals always adds complexity and is not feasible to implement. Additionally, for two-subject concurrent breathing monitoring through the wall Hilbert vibrational decomposition (HVD) technique was integrated which requires synchronous demodulation (Basu et al., 2018). The proposed method can not apply to a single-channel CW radar system as synchronous demodulation is required from the quadrature channel data to integrate the HVD technique. 24-GHz monolithic microwave integrated circuit (MMIC) low-power single-channel radar sensors are the largest transceiver family currently available in the market (Infineon, 2024). Single-channel radar architecture makes the MMIC smaller and more portable (Infineon, 2024; RFbeamMicrowave, 2024). The phase extraction in the single-channel radar module does not necessitate any demodulation algorithms to determine chest displacement information. In contrast, quadrature radar requires the integration of a demodulation algorithm to find the maximum chest displacement information. Moreover, none of the work in the literature focused on exploring the utilization of the single-channel CW radar system for monitoring vital signs such as the heart rate of a single subject and two-subject through the wall. When there is a presence of two subjects through the wall, radar receives interference from heartbeat patterns and isolating independent heartbeat patterns from the mixture is also challenging. This particular work explores the feasibility of utilizing a single-channel 24-GHz on-shelf CW radar system for monitoring the heart rate of a single subject and two subjects through the wall. The core contributions of this work are as follows:

1. The efficacy of the utilization of the single-channel CW radar architecture for monitoring heartbeat through the wall has been tested and showed reasonable accuracy in comparison to the Biopac ECG measurement.
2. The signal processing method MODWT (maximal overlap discrete wavelet transform) has been leveraged for concurrent heartbeat measurement of two subjects through the wall. MODWT method can suppress the subharmonics of the heartbeat signal quite accurately. This method also increased the detection range of the heart rate accuracy of a single subject through the wall.
3. This investigation would help in reducing the number of signal processing chains of the Radar architecture and also leveraging the MODWT method with the single-channel CW Radar system helps to detect the vital signs of two subjects concurrently through the wall.

To the best of our knowledge, this is the first attempt in the literature to utilize single-channel CW radar for extracting the heartbeats of human subjects through the wall both for a single subject and two subjects concurrently. Additionally, the MODWT method has not been used before for isolating the independent heartbeat waveform through the wall. The rest of the paper is organized as follows: the theoretical background of the physiological sensing through the wall is discussed in Section 2. Section 3 illustrates the experimental setup and the results. This section also highlights the comparison of this work with the reported literature.

## 2 Theoretical background

### 2.1 Theory of physiological sensing through the wall

To detect the tiny chest displacement, a CW radar transmits a continuous wave signal that is directed toward the human subject. The phase shift of the reflected echo occurs due to the tiny movement of the chest surfaces (Harikesh et al., 2021). The phase shift of the reflected echo from the tiny movement of the chest surface can be described in Eq. 1 as:

$$\theta \left(t\right)\propto \frac{4\pi }{\lambda }x\left(t\right)$$ (1)

**FIGURE 1:**
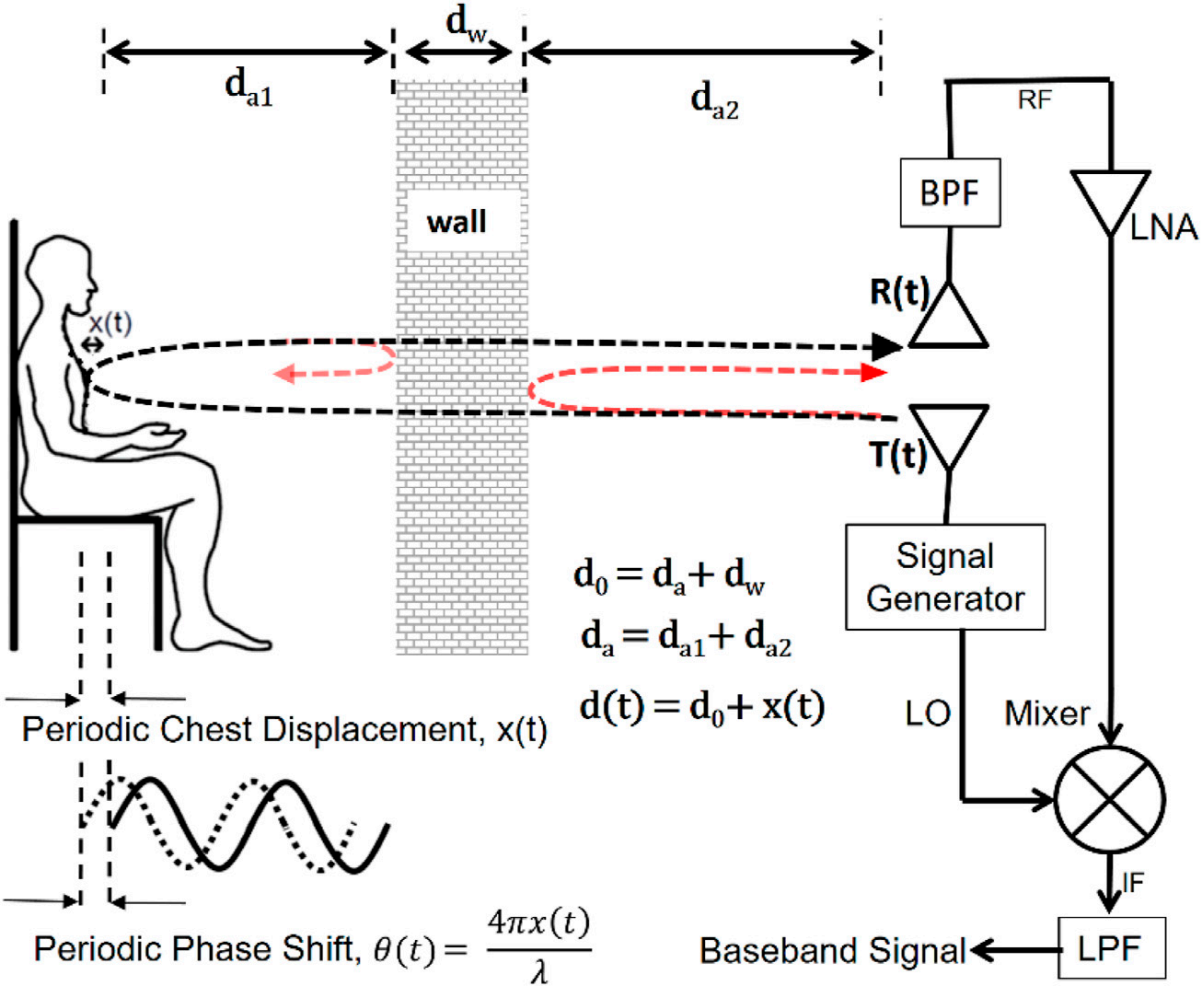
Representation of the theory of physiological sensing through the wall using CW Doppler Radar. Traditionally, a radar system transmits a signal, and the reflected signal is directly proportional to the small chest movements of the chest surface caused by cardiorespiratory activities.

Due to the presence of the wall, the phase-modulated reflected echo experiences attenuation that can be contributed by the depth of the wall, absorption, and reflection of the signal energy as it interacts with the wall material (Carr, 2000) shown in Figure 1. The received signal can be written as:

$$R\left(t\right)=A_{R}\cos\left[2\pi f_{c}\left(t-t_{d}\right)+\varphi \left(t-t_{d}\right)+\theta_{0}\right]$$ (2)

Where, $t_{d}$ is the round trip time delay and $A_{R}$ is the reflected signal amplitude = a×w×c ( a,w,and c are the complex transmission coefficients for the air, wall, and chest respectively). Substituting $t_{d}=\frac{2d\left(t-\frac{d\left(t\right)}{c}\right)}{c}$ in Eq. 2 we get,

$$R\left(t\right)\approx A_{R}\cos\left[2\pi f_{c}t-\frac{4\pi }{\lambda }\left(d_{0}+x\left(t\right)\right)+\varphi \left(t-\frac{2d_{0}}{c}\right)+\theta_{0}\right]$$ (3)

In Eq. 3 $d_{0}$ is the distance between the chest surface and the transmitter antenna ( $d_{a}$ is the distance covered in air, $d_{w}$ is the depth of the wall). Radar receiver consists of mixers which is non-linear (Carr, 2000; Islam et al., 2023). When there is a presence of two subjects in front of the radar, two reflected RF echoes produce an output as expressed in Eq. 4:

$$M_{2}\left(t\right)=\cos\left(2\pi f_{c}t\right)*\left[R_{1}\left(t\right)+R_{2}\left(t\right)\right]$$ (4)

where, $f_{c}$ is the carrier frequency and $R_{1}\left(t\right),R_{2}\left(t\right)$ is the reflected echoes from the two subjects.

$$\begin{array}{ll} M_{2}\left(t\right) & =\cos\left(2\pi f_{c}t\right)*[A_{1}\left(t\right)\cos\left(2\pi f_{c}t-\frac{4\pi }{\lambda }\left(d_{0,1}+x_{1}\left(t\right)\right)\right)+A_{2}\left(t\right)\cos\left(2\pi f_{c}t-\frac{4\pi }{\lambda }\left(d_{0,2}+x_{2}\left(t\right)\right)\right) ]\\ & =\frac{A_{1}\left(t\right)}{2}[\cos\left[\frac{4\pi }{\lambda }\left(d_{0,1}+x_{1}\left(t\right)\right)\right]+\cos\left[4\pi f_{c}t-\frac{4\pi }{\lambda }\left(d_{0,1}+x_{1}\left(t\right)\right)\right] ]+\frac{A_{2}\left(t\right)}{2}[\cos\left[\frac{4\pi }{\lambda }\left(d_{0,2}+x_{2}\left(t\right)\right)\right]+\cos\left[4\pi f_{c}t-\left(d_{0,2}+x_{2}\left(t\right)\right)\right] ] \end{array}$$ (5)

where $A_{n}\left(t\right)$ represents the amplitude modulation of the signal reflected by subject n, $d_{0,n}$ represents the nominal distance to subject n, and $x_{n}$ (t) stands for the n subject’s time-varying physiological displacement. For simplicity, the phase noise of the oscillator (φ) and constant phase shift ( $\theta_{0}$ ) terms are ignored in Eq. 5. The output of the mixer carries modulated components at both sum and difference frequencies. The sum frequency can easily be rejected by a lowpass filter, leaving only the difference frequencies, which are at baseband and shown as B_2_(t) in Eq. 6.

$$B_{2}\left(t\right)=\frac{A_{1}\left(t\right)}{2}\cos\left[\frac{4\pi }{\lambda }\left(d_{0,1}+x_{1}\left(t\right)\right)\right]+\frac{A_{2}\left(t\right)}{2}\cos\left[\frac{4\pi }{\lambda }\left(d_{0,2}+x_{2}\left(t\right)\right)\right]$$ (6)

Due to the non-linear nature of the mixture it produces both the sum and difference frequencies that create an intermodulation tone (Carr, 2000; Islam et al., 2023). For the intermodulation tone, subharmonics would be dominant in the spectrum and would hinder the dominant peaks in the spectrum. Therefore, subharmonics compression is essential to extract the dominant fundamental frequency from the spectrum for multi-subject scenarios.

### 2.2 Maximal overlap discrete wavelet transform (MODWT)

The radar-reflected echoes $y\left(t\right)=B_{2}$ (t) can be divided into two components using the MODWT method (Nguyen, , 2021; Shrifan et al., 2021). One is the detailed component and another is the approximation method shown below in the equation:

$$y\left(t\right)=\sum_{n}c_{j,n}\phi_{j,n}\left(t\right)+\sum_{j=J}^{\infty }\sum_{n}d_{j,n}\psi_{j,n}\left(t\right)$$ (7)

In Eq. 7, the function ϕ(t) is the proportional function of two consecutive signals with the approximate function or the ratio coefficient { $c_{j,n}$ }, and ψ(t) denotes the wavelet function with the wavelet coefficient { $d_{j,n}$ }, represented Eq. 8 as:

$$\begin{array}{c} d_{j,n}=2^{-\frac{j}{2}}\int_{-\infty }^{\infty }y\left(t\right)\psi_{j,n}\left(2^{-j}\left(t-n\right)\right)dt,\\ c_{j,n}=2^{-\frac{j}{2}}\int_{-\infty }^{\infty }y\left(t\right)\phi_{j,n}\left(2^{-j}\left(t-n\right)\right)dt \end{array}$$ (8)

The wavelet coefficient of the wavelet signal and its ratio coefficient are related in the following way:

$$\begin{array}{c} c_{j,n}^{\left(M\right)}=\frac{1}{2^{j/2}}\sum_{-\infty }^{\infty }g_{j,k}y_{n-kmodN,}\\ d_{j,n}^{\left(M\right)}=\frac{1}{2^{j/2}}\sum_{-\infty }^{\infty }h_{j,k}y_{n-kmodN} \end{array}$$ (9)

In Eq. 9, g_j_,_k_ represents the low-pass filter, h_j,k_ is the high-pass filter, k is the discrete wavelet transform to the M^th^ levels, and N is the length of the signal sample. In general, the MODWT multiresolution analysis approach decomposes an original signal into detailed and approximate fields without sampling down in the filtering process to extract the fundamental frequency components and subharmonics or intermodulation-related components (Shrifan et al., 2021).

## 3 Experimental setup and results

### 3.1 Experimental setup

**FIGURE 2:**
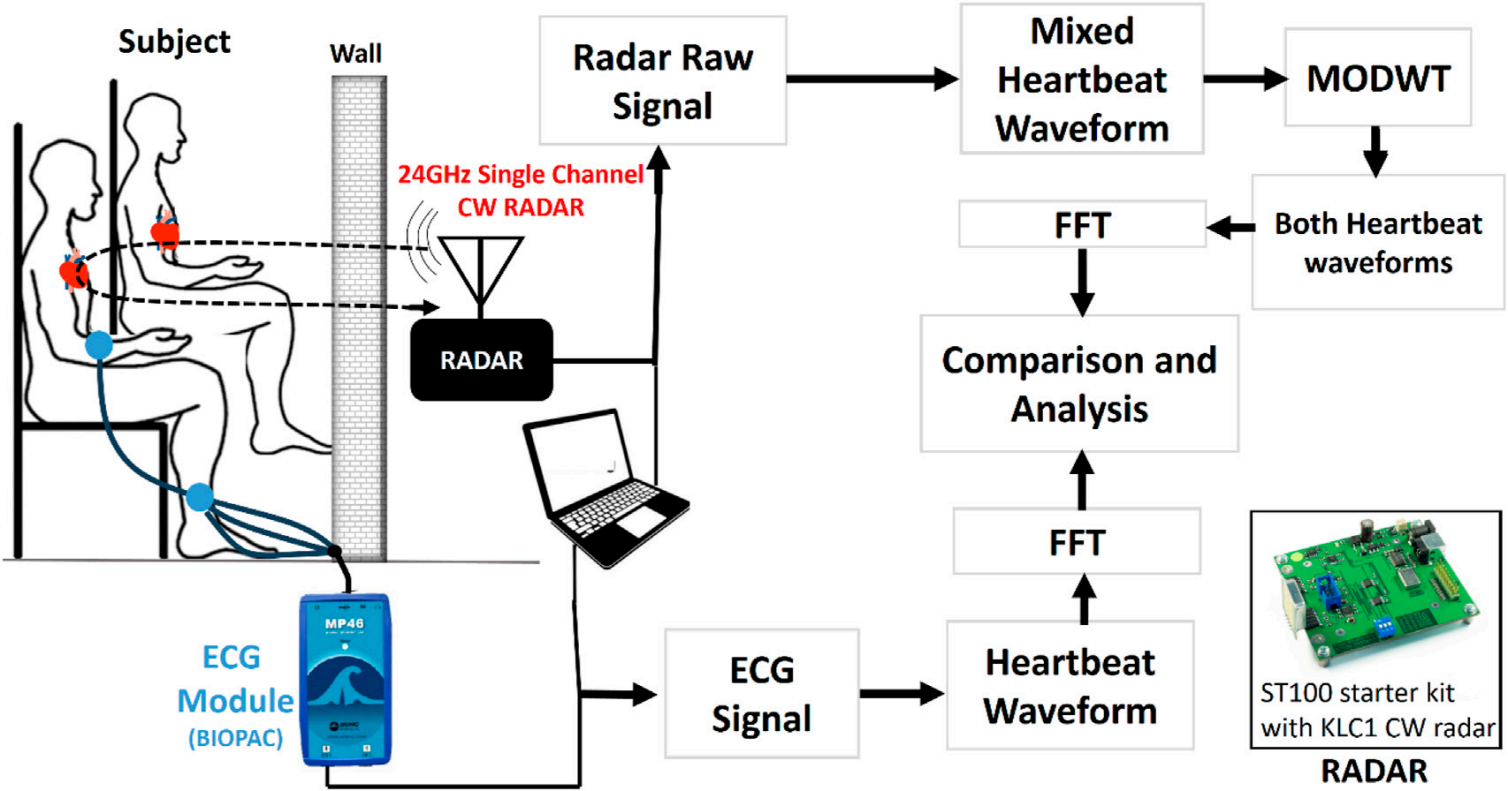
Block diagram of the proposed system. Radar raw signal is captured both for single subjects and concurrent measurements of two subjects. Then using the MODWT method subharmonics suppression is performed to extract the fundamental frequency and then calculated its accuracy with the Biopac ECG acquisition module that acts as a reference system.

**FIGURE 3:**
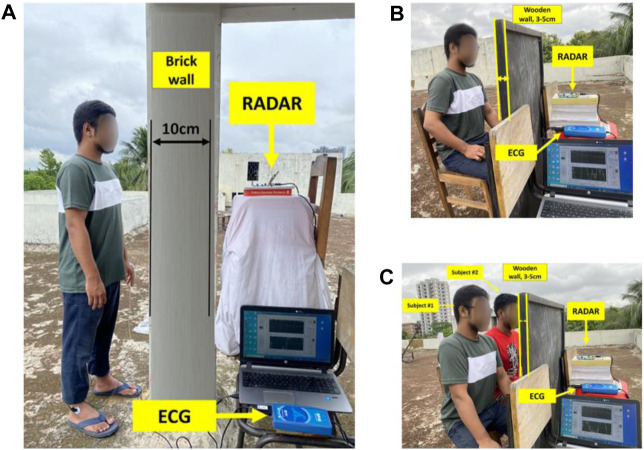
Experimental setup for human heart signal reception through the wall using Doppler Radar. **(A)** Single subject and brick wall **(B)** Single subject and wood wall **(C)** Two concurrent subjects and wood wall.

The proposed system consists of a 24-GHz single-channel on-shelf ST100 starter kit with the KLC1 CW radar module shown in Figure 2. The KLC1 radar transceiver has a single channel with a dual 4-patch antenna with a beam aperture of 80° and 34°, along with an output power of 15dBm (RFbeamMicrowave, 2024). The on-shelf ST100 starter kit with the KLC1 radar module has a graphical user interface (GUI) for data collection and it has a 16-bit low-noise analog-to-digital converter (ADC) that can process signals up to 16 KHz (RFbeamMicrowave, 2024). For this work, we used a sampling frequency of up to 1 KHz for data collection. The experiment was performed in a home environment (not an anechoic chamber) to resemble a life situation shown in Figure 3. We collected the data from ten different healthy subjects with an age group between 21 and 30 in different environments such as a room environment and rooftop of the floor. The participants also do not have any heart disease-related issues. This study was performed with the approval of the ethical review board of the Faculty of Biological Science, University of Dhaka, Dhaka, Bangladesh. During the data collection process, the participants were mostly in sedentary positions without any movements. We also used two different types of barriers such as brick walls and wood between the human subject and the radar system. The thickness of the brick wall is about 10 cm and the thickness of the wooden wall is approximately 5 cm. Each subject was positioned at five different distances ranging from 20 to 100 cm. A total of 35 data sets were collected for single-subject measurements, with 25 sets recorded in the presence of a brick wall and 10 sets with a wood wall. The data recording process was conducted for an approximate duration of 1 min each time. Therefore, 35 min of data, encompassing around 600 breathing and 2,500 heartbeat cycles, was collected. To further investigate, we also collected four data sets for concurrent measurements of two subjects positioned in front of the radar, with a subject-radar distance of 40 cm and a wood wall in between, as illustrated in Figure 3C.

### 3.2 Results

**FIGURE 4:**
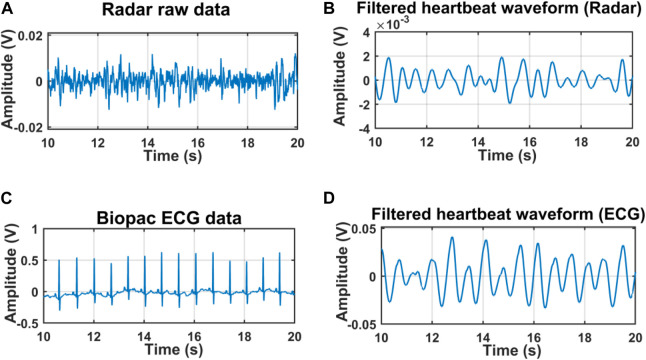
Radar-captured data when the subject was at 40 cm distarnce through the wall and was in seated position. Raw signal **(A)** from Radar **(C)** Biopac ECG acquisition module. Filtered signal **(B)** from Radar, and **(D)** from ECG.

**FIGURE 5:**
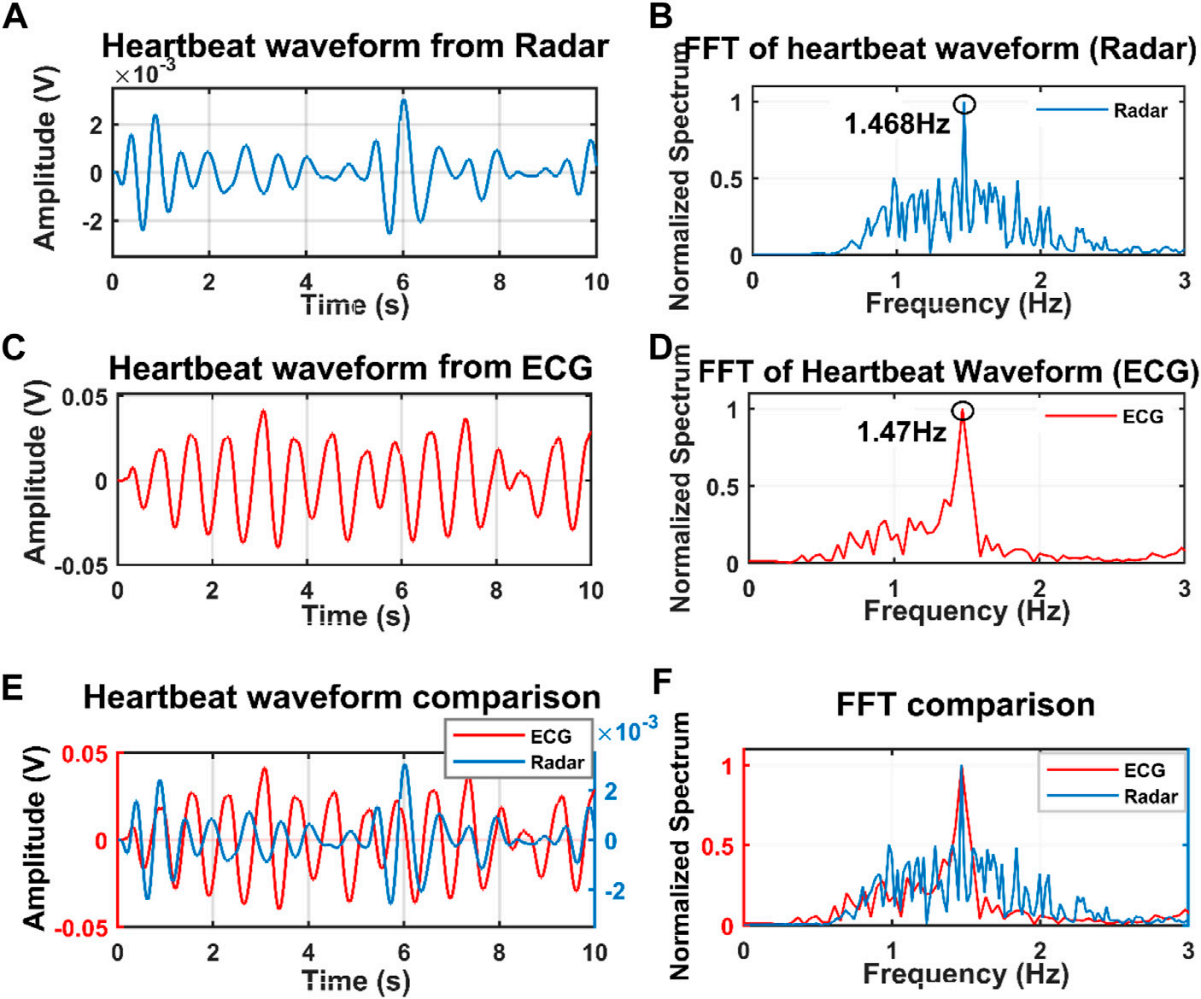
Filtered waveform when the subject was at the distance of 40 cm from the radar through the wall. **(A)** Radar data, and **(C)** Biopac-ECG data. FFT of the radar filtered data **(B)** and ECG waveform filtered data **(D)**. Both the filtered data and FFT of the filtered signal was drawn in time domain **(E)** and frequency domain **(F)**.

**FIGURE 6:**
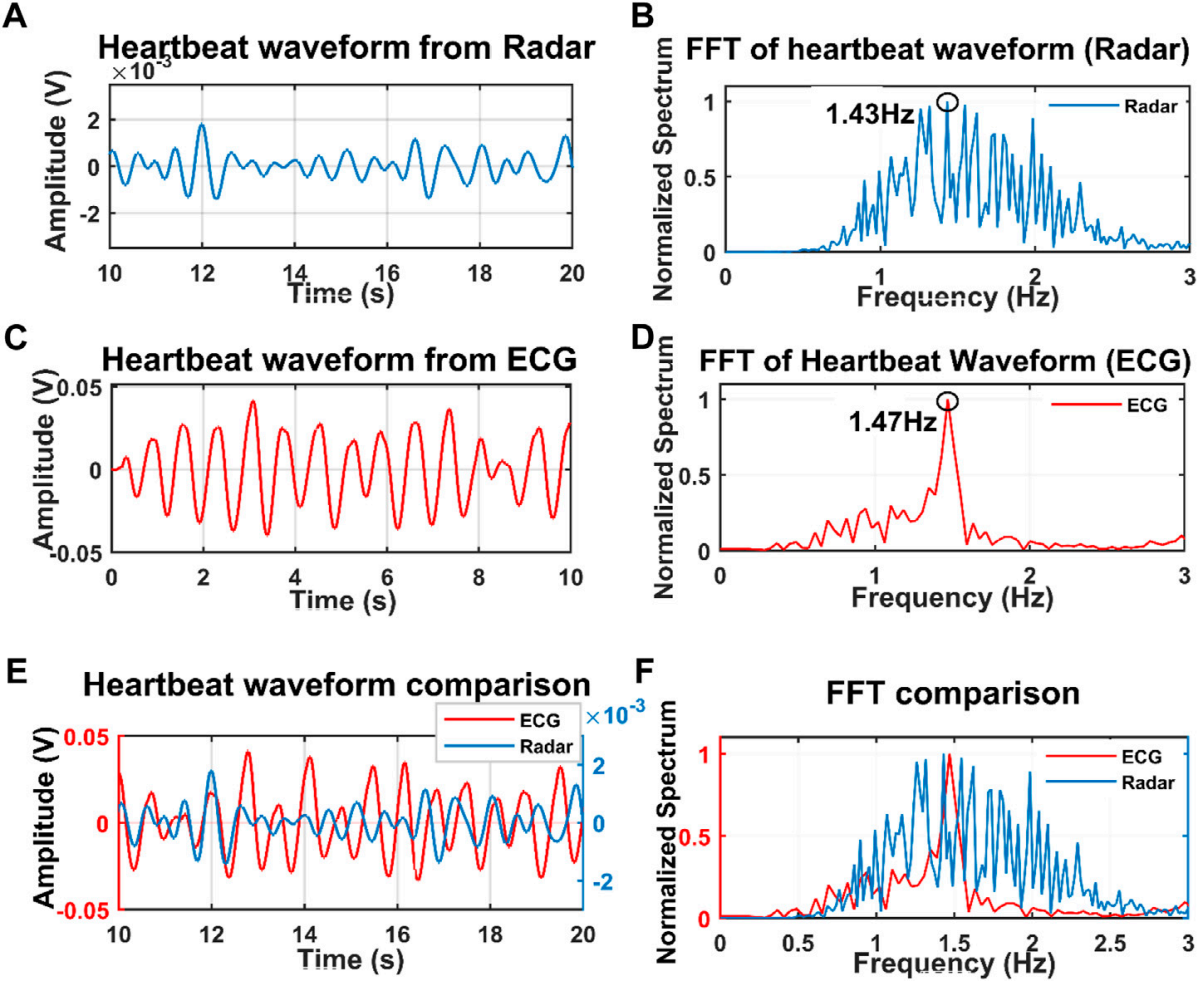
Filtered heartbeat waveform from the radar **(A)** and Biopac ECG acquisition module **(C)** when the subject was at a distance of 100 cm through the wall. FFT of the radar-captured signal **(B)** and ECG waveform shown in **(D)**. Both the time domain and frequency domain of radar-captured and ECG-captured signals are plotted together in **(E)** and **(F)**. The FFT peak from the radar-captured signal can not be determined as the subharmonics are dominant.

**FIGURE 7:**
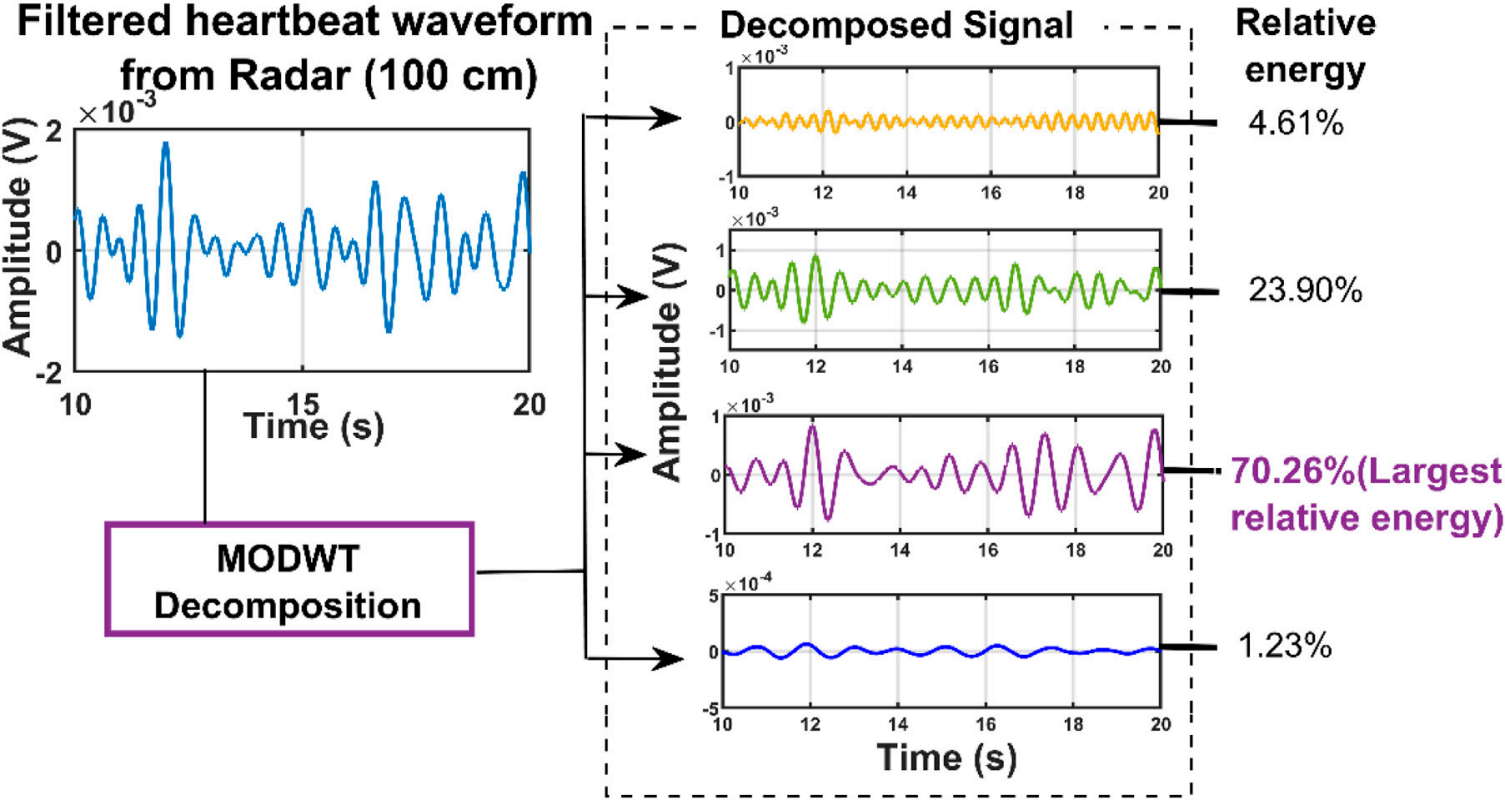
The decomposed signal and its associated relative energy are shown here. The higher relative energy has been selected as the desired heartbeat signal from the Radar.

**FIGURE 8:**
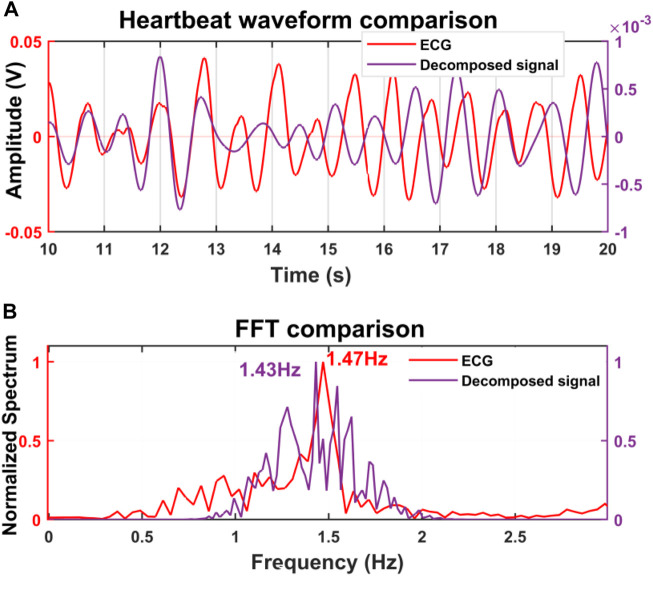
**(A)** Time domain signal of the MODWT-decomposed signal and ECG signal. **(B)** The FFT of the best decomposed signal was 1.43 Hz that closely matched with the FFT of the ECG signal.

For initial verification of the proposed system, we collected data just for single subject presence in front of the radar through the wall and wooden wall as well. The data was collected when the subjects were in seated and standing positions for a distance of 20 cm up to 100 cm Figure 4 illustrates the raw data that was collected when a single subject was in a seated position at a distance of 40 cm from the radar including the wall thickness. After capturing the data we removed the DC portion of the signal by deducting the mean of the signal and then filtered it with the bandpass filter with the cut-off frequency of 0.8–2 Hz for extracting the heartbeat waveform. Figures 4B, D illustrate the filtered heartbeat waveform. After filtering the heartbeat waveform we performed a Fast Fourier transform (FFT) to extract the fundamental frequency of the captured signal which is the heartbeat of the subject. During all the measurements we also collected data using the Biopac ECG acquisition module to compare the accuracy of our proposed system. Figure 5 shows that the radar-extracted heartbeat was 1.468 Hz and the Biopac ECG acquisition module captured heartbeat was around 1.47 Hz. This clearly illustrated that the radar signal extracted heartbeat waveform perfectly matches the gold standard reference system ECG module extracted heartbeat. It has been observed that when a single subject started crossing more than 50 cm the radar-captured signal strength started degrading and subharmonics became very dominant so that the peak of the FFT can not be determined as other subharmonics are very strong. Figure 6 clearly shows that the FFT of the radar-captured signal does not match the Biopac ECG acquisition module. By leveraging the MODWT method we could extract the heartbeat-related information even at distances of 80 and 100 cm. MODWT method decomposes the signal into four different levels based on the frequency range of the decomposition level (MathWorks, 2024). Additionally, it identifies the relative energy of the signal at multiple levels. Figure 7 illustrates that the radar-captured signal at a distance of 100 cm is being decomposed into four different levels and its associated relative energy has been calculated and shown in Figure 7. The highest relative energy decomposed signal was selected as the heartbeat signal and then its FFT was compared to the FFT of the Biopac ECG acquisition module. Figure 8 illustrates the time domain signal of the MODWT-decomposed best signal, the ECG signal, and then its associated FFT was also illustrated. From the FFT of the MODWT decomposed best signal it is visible that the subharmonics compression was performed accurately. For the subharmonics compression using the MODWT method, the FFT of the radar-captured signal showed 1.43 Hz whereas the ECG signal showed 1.47 Hz. That clearly illustrated the efficacy of the MODWT method.

**FIGURE 9:**
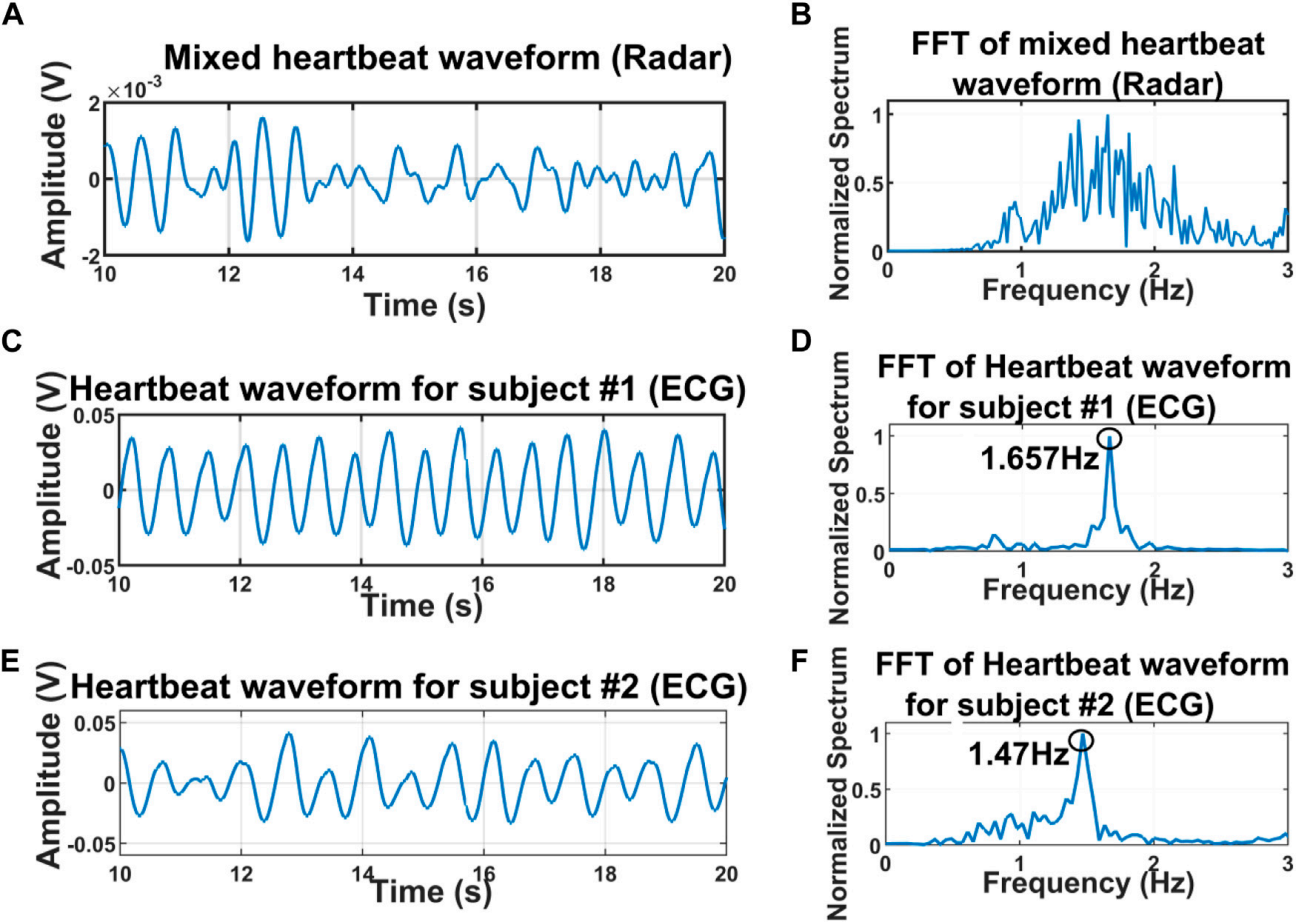
Time domain signal of the mixture of two subjects at a distance of 40 cm through the wall **(A)** and its associated FFT **(B)**. The ECG signal of the individual subject 1 **(C)** and subject 2 **(E)** and the FFT of the signal denoted that the heart rate of subject-1 was 1.657 Hz **(D)** and 1.47 Hz **(F)**.

**FIGURE 10:**
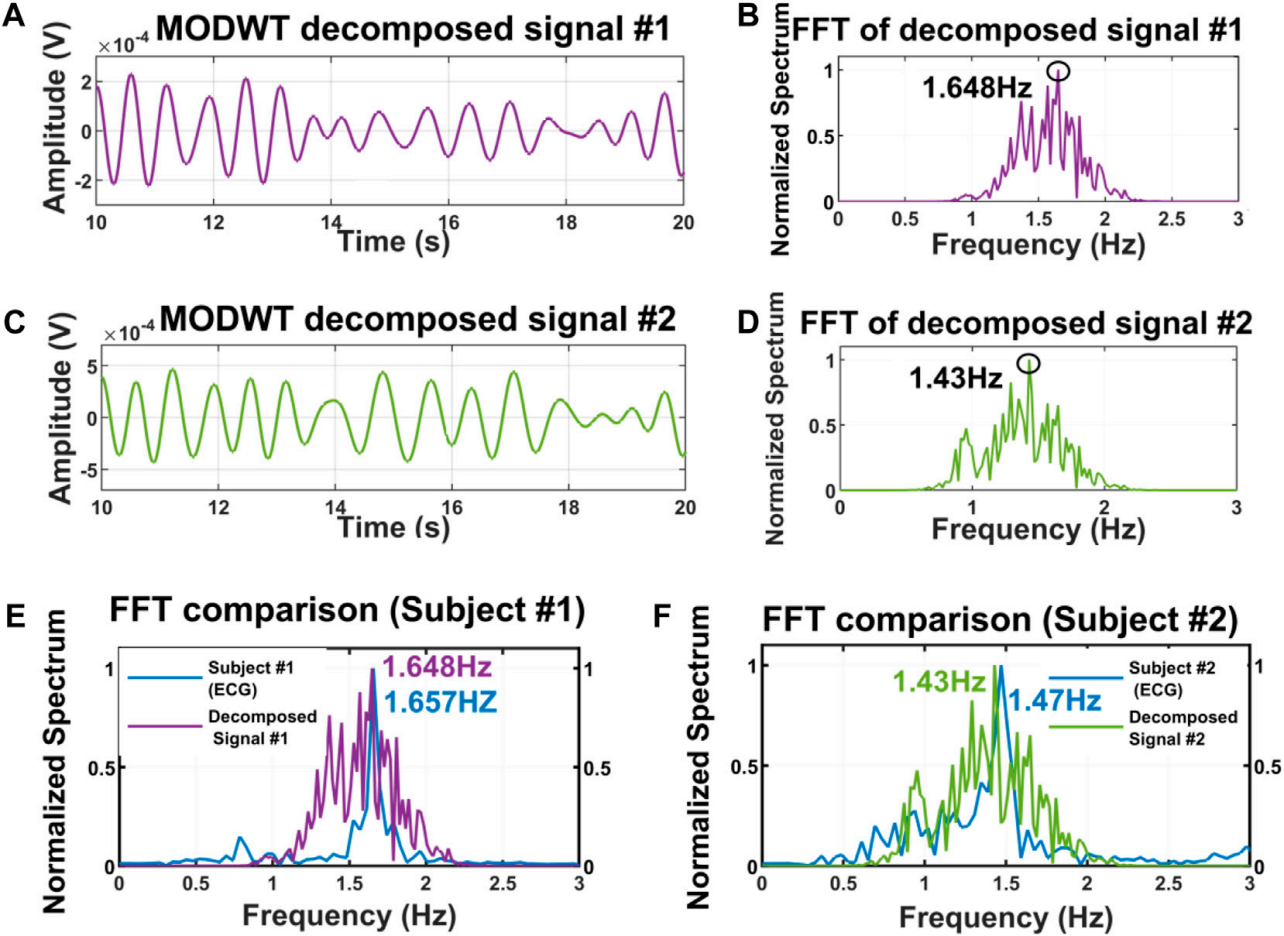
Time domain signal of the MODWT decomposed best two signals **(A)**, **(C)** and its associated FFTs **(B)**, **(D)**. The FFT comparison between the best decomposed signals and ECG signal of subject 1 **(E)** and subject 2 **(F)** closely matches each other.

We also experimented with the presence of the two subjects in front of the radar through the wood as well. The distance of the subject from the radar was approximately 40 cm from the human and the shoulder-to-shoulder distance between the subjects was almost 10 cm Figure 9 illustrates that when there is a presence of two subjects through the wall, radar receives multiple reflections and echoes from the target and therefore, isolating the independent respiratory patterns from the combined mixture is always a challenging task (Islam et al., 2022a). The FFT of the mixture signal shows multiple peaks and the fundamental frequency can not be determined due to the presence of the other dominant peaks shown in Figure 9. Figures 9C, E illustrates the ECG signal of individual subjects 1, and 2 and its associated FFTs (d) and (f). Then the MODWT method was applied to the mixture of the signal to isolate the independent respiratory patterns. Figure 10 illustrates the two best-decomposed MODWT signals that were selected based on the relative energy of the signal. After decomposition and selecting the best two signals, we performed the FFT. The FFT peak of the best two decomposed signals is 1.648 and 1.43 Hz which quite accurately matches with the ECG signal.

**FIGURE 11:**
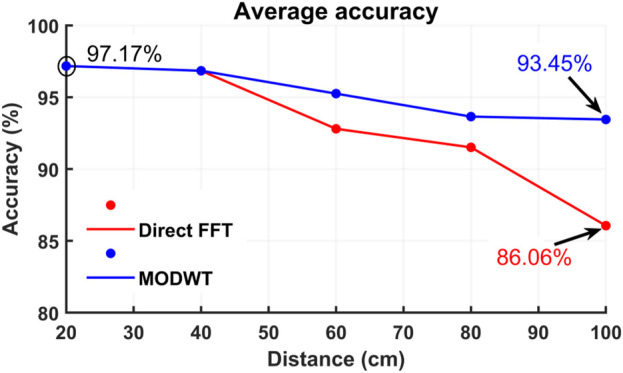
The comparative average accuracy of the direct FFT method and MODWT method at different distances of the subjects through the wall. MODWT and direct FFT methods perform quite closely up to distances of 40 cm and showed a similar accuracy of 96.84%. At a distance of 60 cm, the direct FFT method accuracy falls to 92.80% whereas the accuracy of the MODWT method is almost 95.25%. For the distance of 100 cm, the direct FFT method accuracy degrades to 86.06% and the accuracy of the MODWT method is still very high showing an accuracy of 93.45%.

To test the reproducibility and repeatability of the proposed system, we collected a dataset from seven participants for single-subject and eight participants for two-subject concurrent measurements, having distances from 20 cm to up to 100 cm. We also performed the typical FFT method for extraction of the heart rates through the brick wall and the wooden barrier as well. Table 1 illustrates the overall accuracy investigation for the single subject and Table 2 shows the accuracy investigation for two-subject concurrent measurement. The direct FFT method demonstrated reasonable accuracy up to 60 cm (92.25% at 60 cm) but experienced a decline, dropping to 86.6% at 100 cm distance. On the other hand, the proposed MODWT method showed a better accuracy of 95.25% at 60 cm and 93.45% at a distance of 100 cm, which is reasonable for applications like post-disaster and rescue operations. For the post-disaster search and rescue applications the proposed technology can be the key for saving the lives of trapped and injured people. The direct FFT method can not be implemented for two subjects’ concurrent measurements as it can not isolate the vital signs information for two subjects. However, the MODWT method can also be used for two-subject measurement as it can isolate the heart rate of individual subjects from the multiple echoes. The overall accuracy for two-subject measurements was approximately 97.04%. It is important to highlight that, for two-subject concurrent measurements, four trials were conducted with the radar positioned 40 cm from the human subjects. The accuracy achieved in this scenario closely matches that of the single-subject scenario (96.84% at 40 cm). Figure 11 illustrates the comparative average accuracy of the direct FFT and MODWT methods. The results indicated the efficacy of the proposed MODWT method both for single-subject and two-subject measurements. Using the MODWT method, we could only extract the heart rates of two individuals from their mixed signals. Moreover, the system can detect vital signs up to 1 m accurately, facing challenges beyond this distance in accurately extracting heart rates.

**TABLE 1:** Heart rate accuracy investigation for single subject, using method: Direct-FFT and method: MODWT.

Subject	Gender	Age	Height (cm)	Weight (kg)	Distance (cm)	Wall	HR from ECG (bpm)	HR from radar: Direct-FFT (bpm)	Accuracy: Direct-FFT (%)	HR from radar: MODWT (bpm)	Accuracy: MODWT (%)
#1	M	22	177.8	70	20	Wood	88.2	87.2	98.87	87.2	98.87
					40			88.08	99.86	88.08	99.86
					60			90.0	97.96	90.0	97.96
					80			82.8	93.88	82.8	93.88
					100			85.8	97.28	85.8	97.28
#2	M	24	165.1	67	20	Brick	99.4	97.5	98.09	97.5	98.09
					40			98.8	99.40	98.8	99.40
					60			104.6	94.77	104.6	94.77
					80			89.4	89.94	89.4	89.94
					100			116.4	82.90	101.3	98.05
#3	M	21	170.2	62	20	Wood	78.1	79.3	98.46	79.3	98.46
					40			81.3	95.90	81.3	95.90
					60			85.8	90.14	85.8	90.14
					80			88.5	86.68	72.6	94.50
					100			89.4	85.53	83.5	94.51
#4	M	23	170.2	90	20	Brick	82.3	79.3	96.35	79.3	96.35
					40			85.7	95.87	85.7	95.87
					60			90.2	90.40	87.0	95.29
					80			93.8	86.03	89.2	93.11
					100			96.2	83.11	92.9	89.39
#5	F	26	160.0	56	20	Brick	68.6	73.5	92.86	73.5	92.86
					40			62.5	91.11	62.5	91.11
					60			58.8	85.71	66.6	98.00
					80			68.8	99.71	68.8	99.71
					100			82.6	79.59	72.6	96.00
#6	M	24	180.3	72	20	Brick	90.1	87.2	96.78	87.2	96.78
					40			90.2	99.89	90.2	99.89
					60			94.0	95.67	94.0	95.67
					80			98.0	91.23	98.0	91.23
					100			103.6	85.02	101.6	88.50
#7	M	23	177.8	80	20	Brick	99.4	98.2	98.79	98.2	98.79
					40			95.3	95.88	95.3	95.88
					60			94.4	94.97	94.4	94.97
					80			92.6	93.16	92.6	93.16
					100			88.5	89.03	89.8	90.46

$\text{Accurancy}\left(\%\right)=\left(1-\left|\frac{ECG-RADAR}{ECG}\right|\right)\times 100$ . Each time, the data was recorded for approximately 1 min duration.

**TABLE 2:** Heart rate accuracy investigation for two subjects, from concurrent heartbeat waveform.

Measaurement number	Subjects (concurrent)	Gender	Age	Height (cm)	Weight (kg)	Distance (cm)	Wall	HR from ECG (bpm)	HR from radar (bpm)	Accuracy (%)
1	#1	M	24	165.1	67	40	Wood	99.4	98.8	99.40
	#2	M	22	177.8	70	40		88.2	85.8	97.28
2	#3	M	23	170.2	90	40	Wood	82.3	87.9	93.19
	#4	M	24	160.0	67	40		68.9	67.4	97.82
3	#5	M	23	177.8	80	40	Wood	99.5	102.0	97.48
	#6	M	21	170.2	62	40		78.1	76.2	97.56
4	#7	M	25	162.5	65	40	Wood	92.5	91.8	99.24
	#8	M	28	178.0	82	40		60.2	63.6	94.35

## 4 Comparison with the state-of-the-art

We also compared our proposed method’s success with the existing reported literature. We found in the literature that most of the attempts to measure vital signs through the wall utilized either FMCW (Alizadeh et al., 2019; Liu and Liu, 2014; Wang et al., 2014b; Adib et al., 2015) or UWB radar (Wang et al., 2012; Liang et al., 2018). The reason for utilizing FMCW radar or UWB radar is that it can estimate the range of the target and for multiple subject measurements range azimuth plane determination is very critical (Liu and Liu, 2014). None of the work in the literature focused on utilizing single-channel CW radar for monitoring vital signs through the wall. In the most recent attempt (Harikesh et al., 2021), CW radars were employed by integrating two different frequencies that were transmitted and received simultaneously to measure the range of the target. However, using two different frequencies of antennas would always add complexity, and they also used four different channels for estimating the angular location of the target. All the results either used the direction of arrival (DOA) technique or the direct FFT method for extracting vital signs. In this investigation, we demonstrate that using single channel CW single frequency radar monitoring of vital signs through the wall is possible by leveraging the MODWT method. This is also the first reported result on using single-channel, single-carrier frequency CW radar for monitoring vital signs through the wall. Table 3 illustrates the comparative analysis of the state-of-the-art through-the-wall vital signs monitoring. Single-channel, single-carrier frequency CW radar is simple to implement and can also be used in portable systems such as drones. Additionally, by integrating the MODWT method, two-subject concurrent life signs can also be detected.

**TABLE 3:** Comparison with the state-of-the-art.

Reference and year	Type	Frequency (GHz)	No. of channel	Method	Measured vital signs	No of concurrent subjects	HR accuracy (%)
Harikesh et al. (2021) and 2020	CW	2.49	4	DOA	RR	2	—
Wang et al. (2012) and 2012	UWB	1.5–4.5	1	DOA	RR	2	—
Alizadeh et al. (2019) and 2019	FMCW	77	4	Direct-FFT	HR and RR	1	80%
Liu and Liu (2014) and 2014	SFCW	0.3–1.3	1	Direct-FFT	HR and RR	2	87%
Wang et al. (2014b) and 2014	Hybrid FMCW	5.8 GHz	1	Micro-Doppler	HR and RR	1	94.5%
Adib et al. (2015) and 2015	FMCW	5.46–7.25	1	Vital-Radio	HR and RR	1	90.1%
Liang et al. (2018) and 2018	UWB	0.4	1	FA	RR	1	—
This work	CW	24	1	MODWT	HR	2	97.04%

## 5 Conclusion

Through the wall, human subject heart rate detection using single-channel CW radar has been demonstrated in this paper. The fundamental goal of this work is to test the feasibility of utilizing single-channel CW radar for monitoring the heartbeat of humans through the wall. Additionally, we also tested the feasibility of utilizing CW radar for isolating the heartbeat of two-subjects concurrent measurements in a short-scale study. CW Radar can not find the location of the target, whereas FMCW radar can find the location of the target. However, CW radar has been employed in our other attempts to identify individual human subjects from cardio-respiratory breathing dynamics-related features (Islam et al., 2022b), and that remains our future work to explore. MODWT method demonstrated that without estimating the angular location of the subject, isolation of the individual heartbeat pattern from the combined mixture is also possible with an accuracy of 97.04%. Furthermore, leveraging the MODWT method also increased the accuracy of heartbeat detection after the distances of 40 cm, as the traditional FFT method can only extract heart rate accurately up to 40 cm. The system demonstrated capability in detecting vital signs with reasonable accuracy up to a 1-meter distance. However, beyond this range, the system struggles to extract heart rate accurately. Moreover, the MODWT method enabled us to extract the heart rate of only two individuals from their mixed signals. The proposed simplified architecture of single-channel CW radar, combined with MODWT, holds great promise for various applications, including post-disaster search and rescue, emergency evacuation, security, surveillance, and patient vital signs monitoring. The main strength of single-channel CW radar is the reduction of signal processing components, and it also requires less signal processing chain than multi-channel radar with DOA estimation capabilities. Notably, the simplicity of the single-channel CW radar makes it suitable for portable drone systems, offering potential applications in drone-based life sign detection. Our future investigations will focus on addressing complexities related to motion mitigation in moving drones, aligning with our ongoing goal. Additionally, our research will extend to explore heartbeat detection for more than two individuals simultaneously.

## Data Availability

The raw data supporting the conclusion of this article will be made available by the authors, without undue reservation.
